# Rho-Kinase Inhibitors: The Application and Limitation in Management of Glaucoma

**DOI:** 10.3390/biomedicines13081871

**Published:** 2025-08-01

**Authors:** Yuan-Ping Chao, Ta-Hung Chiu, Da-Wen Lu

**Affiliations:** 1 Department of Ophthalmology, Kaohsiung Armed Forces General Hospital, Kaohsiung 802301, Taiwan; julia206789@gmail.com; 2 Department of Ophthalmology, Tri-Service General Hospital, National Defense Medical University, Taipei 114202, Taiwan; 3 Department of General Medicine, Tri-Service General Hospital, National Defense Medical University, Taipei 114202, Taiwan; dennis963321@yahoo.com.tw

**Keywords:** glaucoma, rho kinase inhibitors, efficacy, side effects

## Abstract

Glaucoma is recognized as a progressive optic neuropathy and a leading cause of irreversible blindness worldwide. While intraocular pressure (IOP) is considered the only modifiable risk factor, current medical treatments are challenged by issues such as inadequate IOP control and ocular side effects. Rho kinase (ROCK) inhibitors have been developed as a novel pharmacologic class targeting the trabecular meshwork to enhance conventional aqueous humor outflow. In this review, the pharmacokinetics and IOP-lowering efficacy of key ROCK inhibitors are summarized. Beyond IOP reduction, ROCK inhibitors exhibit neuroprotective, anti-inflammatory, antifibrotic, and ocular perfusion-enhancing effects. Finally, we analyzed the limitations and future prospects of ROCK inhibitors in the management of glaucoma.

## 1. Introduction

Glaucoma is recognized as a chronic, progressive, degenerative disease that causes retinal ganglion cell loss and optic nerve damage. It is considered the leading cause of irreversible blindness worldwide [1]. From the 2010s to 2040s, the prevalence of glaucoma is estimated to increase substantially, with global projections rising from 64.3 million affected individuals in 2013 to an estimated 111.8 million by 2040 [2]. The etiology of glaucoma is characterized as multifactorial, with contributing factors including age, intraocular pressure (IOP), systemic illness, and genetic predisposition [3]. Among these, IOP is regarded as the only modifiable risk factor. Without timely intervention, glaucoma can lead to rapid visual field deterioration and eventual blindness [4]. The primary goal of treatment is defined as reducing IOP and thereby slowing disease progression [4,5]. Management of glaucomatous optic neuropathy is complex and continually evolving, encompassing pharmacological therapy, laser procedures, and surgical interventions [6,7]. Pharmacological therapy remains the primary noninvasive treatment approach for glaucoma.

The first reported use of medication for glaucoma was documented in the 1870s, when cholinergic agonists were first employed [8]. Over time, various drug classes, including beta-blockers, carbonic anhydrase inhibitors, alpha-2 agonists, and prostaglandin analogs have been introduced. Topical ocular hypotensive medications are broadly categorized into two groups: those that reduce aqueous humor production and those that enhance aqueous outflow. Beta-blockers, carbonic anhydrase inhibitors, and alpha-2 agonists decrease aqueous production within the ciliary body. In contrast, cholinergic agonists increase outflow by inducing morphological changes in the irido-corneal angle, while prostaglandin analogs increase uveoscleral outflow [9,10]. Despite a wide range of options on topical ocular hypotensive medication, none targeted the conventional aqueous outflow pathway until the emergence of Rho kinase (ROCK) inhibitors.

**Figure 1:**
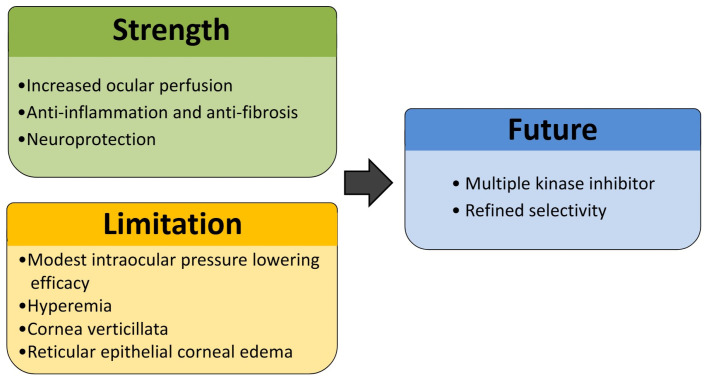
This diagram summarizes three key aspects of Rho kinase inhibitors. Strengths include their ability to enhance ocular perfusion, modulate postoperative fibrosis and inflammation, and provide neuroprotection. Limitations involve modest IOP-lowering efficacy and ocular side effects. Future directions focus on the development of multiple kinase inhibitors with enhanced selectivity to improve therapeutic outcomes and minimize side effects.

ROCK is a serine/threonine-specific protein kinase. It has been demonstrated that the Rho/ROCK pathway plays a unique role in the trabecular meshwork (TM) by affecting actomyosin cytoskeletal structure and extracellular matrix (ECM) formation. ROCK inhibitors promote relaxation of the TM by attenuating actin cytoskeletal contractility, facilitate nuclear translocation of β-catenin to suppress cellular migration, interfere with ATP-dependent phosphorylation events to inhibit ROCK1 and ROCK2 activity, and prevent the association of Rho GTPases with ROCK [11]. Aqueous humor is known to exit the anterior chamber via TM but also encounter outflow resistance in TM [12]. Increased TM cell contractility and ECM accumulation are observed to narrow intercellular spaces, raising outflow resistance and increasing IOP. ROCK inhibitors are used to relax TM cells and inhibit ECM deposition, thereby facilitating aqueous humor outflow [13,14,15]. Ripasudil and netarsudil are two topical ROCK inhibitors approved for glaucoma treatment [16,17]. Multiple pivotal trials have been conducted to substantiate the IOP-lowering efficacy. Ripasudil was shown to demonstrate significant reductions in IOP compared to the baseline across phase II and III studies in patients with primary open-angle glaucoma (POAG) and ocular hypertension (OHT), both as monotherapy and in combination with timolol or latanoprost [18,19,20]. Similarly, netarsudil 0.02% once daily was found to achieve clinically significant IOP reduction in the ROCKET-1 and ROCKET-2 phase III trials [21]. Ripasudil (Glanatec ophthalmic solution 0.4%; Kowa Company, Ltd., Japan) is approved and marketed in Japan, South Korea, Singapore, and several other East and Southeast Asian countries. However, ripasudil has not been submitted for regulatory approval in Western countries or Taiwan. Conversely, netarsudil (Rhopressa; Aerie Pharmaceuticals, USA) has obtained regulatory approval for the reduction of elevated IOP in the United States and European Union. In addition to their IOP-lowering effects, ROCK inhibitors are known to exhibit several additional pharmacologic effects. This article aims to review pharmacokinetics, effectiveness, anatomical and functional changes, side effects, and the prospect of ROCK inhibitors (Figure 1).

## 2. Pharmacokinetics and Intraocular Pressure-Lowering Profiles of Marketed Rho Kinase Inhibitors

### 2.1. Netarsudil (AR-13324)

#### 2.1.1. Pharmacokinetics

Netarsudil is a dual mechanism compound that functions both as a ROCK inhibitor and norepinephrine transporter inhibitor. It demonstrates high ocular bioavailability with minimal systemic absorption. Following topical administration in rabbits, systemic tissue radioactivity (blood and plasma) was found to be 200–3000 times lower than in ocular tissues. The peak concentration (T_max_) in aqueous humor is observed to occur approximately 8 h post-administration. The drug is primarily distributed in the cornea and conjunctiva, followed by the iris/ciliary body, retina–choroid complex, and aqueous humor, with negligible concentrations in the vitreous and lens. Netarsudil is metabolized by esterases in the cornea to form its active metabolite, netarsudil-M1, which exhibits potent ROCK inhibition and is the dominant form detected in aqueous humor. The conversion of netarsudil to netarsudil-M1 represents optimal pharmacokinetic design, as this metabolite becomes the dominant form in aqueous humor and exhibits enhanced ROCK inhibition compared to the parent compound, ensuring maximum pharmacodynamic effect at the site of action. Variable tissue-specific elimination is demonstrated; half-lives of 12–27 h are reported in blood, plasma, and cornea, whereas prolonged half-lives up to 112 h are observed in the iris/ciliary body, retina–choroid complex, and lens [22].

#### 2.1.2. IOP Lowering Efficacy

Netarsudil is typically administered once daily (q.d.), although twice daily (b.i.d.) dosing has been explored in some studies. IOP reductions of 16–22% (q.d.) and 22–24% (b.i.d.) have been reported with 0.02% netarsudil. The mean IOP reduction is documented to be approximately 3.3–4.7 mmHg (q.d.) and 4.1–5.4 mmHg (b.i.d.) [14,23,24]. When combination therapy is employed, an additional 2.2–3.3 mmHg reduction is achieved by netarsudil 0.02%/latanoprost 0.005% compared to netarsudil monotherapy [25]. The IOP-lowering effect is primarily attributed to an enhancement in trabecular outflow facility, which increased by 22%, along with a 10% reduction in episcleral venous pressure [26]. In individuals already receiving a quadruple regimen of IOP-lowering agents—including prostaglandin analog, beta-blocker, carbonic anhydrase inhibitor, and alpha-agonist—the addition of netarsudil has been shown to further reduce IOP by 4.0 ± 1.9 mmHg [27].

### 2.2. Ripasudil (K-115)

#### 2.2.1. Pharmacokinetics

High concentrations are achieved by ripasudil in the cornea, conjunctiva, and anterior chamber tissues, with minimal systemic distribution being observed. It is rapidly absorbed, achieving peak plasma concentration (T_max_) within 0.08–0.30 h after topical administration. A half-life of approximately 0.6–0.7 h is exhibited by ripasudil, while its active metabolite (M1), which is generated via aldehyde oxidase, demonstrates a longer half-life of 2.0–2.6 h. Ripasudil, compared to netarsudil, demonstrates rapid absorption with a shorter T_max_ and shorter elimination half-life, necessitating twice-daily administration to achieve optimal IOP reduction. The drug is predominantly eliminated via hepatic metabolism [28].

#### 2.2.2. IOP Lowering Efficacy

- POAG and OHT: In the prospective observational J-ROCK study and a randomized controlled trial by the K-115 Clinical Study Group, IOP reductions of 3.0–4.5 mmHg over 4 to 8 weeks were achieved by ripasudil 0.4% b.i.d. [14,18]. When employed as an adjunctive treatment with other glaucoma medications, IOP was reduced by 2.6–2.9 mmHg with ripasudil b.i.d. [29,30].
- Secondary glaucoma: In the ROCK-S retrospective cohort study by Futakuchi et al. [31], 332 eyes with secondary glaucoma (including uveitic, exfoliative, and steroid-induced glaucoma) were treated with 0.04% ripasudil. Statistically significant IOP reduction was observed at 1, 2, and 6 months of follow-up, with a maximal reduction of 7.00 ± 8.60 mmHg being achieved at 6 months.

### 2.3. Fasudil

#### 2.3.1. Pharmacokinetics

Fasudil is a hydrophilic compound with a low molecular weight (MW 327.83 g/mol), resulting in low entrapment efficiency and increased drug leakage when used with ocular drug carriers. Consequently, poor ocular bioavailability (0.1–5%) is demonstrated by this compound [32]. Minimal systemic absorption is expected due to local metabolism or tear drainage. While fasudil is not approved for eye diseases, it is frequently studied in ophthalmic research.

#### 2.3.2. IOP Lowering Efficacy

Despite these pharmacokinetic limitations, the limited clinical evidence available demonstrates that fasudil retains pharmacodynamic activity when adequate concentrations are achieved. In an interventional case series, four eyes with end-stage POAG were treated with topical fasudil b.i.d. Mean IOP reductions of 8.25 ± 1.2 mmHg at 2 h and 8.75 ± 2.2 mmHg at 4 h post-administration were observed [33].

## 3. Role of Rho Kinase Inhibitors in Ocular Perfusion

In animal studies, increased velocity and volume of optic nerve head perfusion were observed following topical administration of ROCK inhibitors, such as ripasudil and Y-39983 [15,34]. More invasive administration routes, including intravitreal injection of ripasudil and intravenous infusion of fasudil, were associated with dilation of the retinal arteries. These effects are believed to result from ROCK-inhibitor-mediated relaxation of vascular smooth muscle via calcium sensitization, facilitating blood flow [35,36]. Increased ocular perfusion is hypothesized to contribute to the neuroprotective potential of ROCK inhibitors. However, the precise mechanisms linking vascular modulation to neuroprotection remain unclear.

## 4. Modulation of Inflammation and Fibrosis by Rho Kinase Inhibitors

Trabeculectomy remains a cornerstone surgical procedure for reducing IOP by enhancing aqueous outflow. However, postoperative inflammation and fibrosis, particularly involving Tenon’s capsule and conjunctival tissues, are major causes of surgical failure [37,38]. Various agents, including intraoperative and postoperative mitomycin-C, 5-fluorouracil, and corticosteroids, are routinely used to modulate wound healing [39,40,41,42,43]. The concentration of pro-inflammatory cytokines in the aqueous humor has been identified as a risk factor not only for surgical failure, but also for the progression of POAG and neovascular glaucoma [44]. ROCK inhibitors have demonstrated the capacity to modulate inflammatory cytokine activity and fibrosis formation. Among these, the transforming growth factor-beta (TGF-β) pathway is considered of particular relevance [45]. TGF-β1-induced myofibroblast transdifferentiation in human conjunctival fibroblasts was inhibited by AR12286 [45]. TGF-β1-induced Smad2/3 signaling in Tenon’s capsule fibroblasts was suppressed by Y-27632 [46] (Table 1). The influence of ROCK inhibitors on other cytokines, such as interleukin-6 (IL-6) and IL-8, has been minimally reported. Although the anti-inflammatory and antifibrotic potential of ROCK inhibitors is supported by in vitro and animal studies, clinical studies have provided limited evidence to confirm these effects in surgical success. In a randomized clinical study comparing ripasudil-treated and non–ripasudil groups, no significant differences in IOP were observed following trabeculectomy augmented with mitomycin C in patient with open angle glaucoma [47]. Nevertheless, the postoperative use of ripasudil was associated with a significant reduction in the number of IOP-lowering medications required during the follow-up period. Similarly, in a cross-sectional study by Mizuno et al. [48], the addition of ripasudil after a needling procedure with mitomycin C for failed trabeculectomy did not improve IOP outcomes or surgical success rates compared to mitomycin C alone. These findings suggest that the translation of its antifibrotic effects from preclinical models to clinical outcomes remains uncertain.

Corticosteroids are widely used across various disease indications to control inflammation. However, corticosteroid-induced IOP elevation is a well-recognized adverse effect. The underlying mechanisms involve disruption of mucopolysaccharide metabolism, enhanced ECM production, and reduced phagocytic activity of TM cells. Corticosteroids cause excessive material accumulation within outflow channels and increased outflow resistance [49].

Autotaxin–lysophosphatidic acid (ATX–LPA) signaling has been implicated as an upstream regulator of ROCK activation in the TM [50]. Autotaxin (ATX), a secreted glycoprotein with lysophospholipase D activity, generates LPA from lysophosphatidylcholine in the aqueous humor. LPA subsequently binds to lysophosphatidic acid receptors (primarily LPAR1 and LPAR3) on TM cells, triggering downstream RhoA activation and ultimately leading to ROCK pathway stimulation. Upon stimulation by factors such as dexamethasone, TM cells significantly upregulate ATX expression and secretion, leading to increased local LPA production. LPA activates downstream RhoA signaling and ultimately the ROCK pathway. This ATX–LPA–RhoA–ROCK cascade promotes cell contractility, fibrotic changes, and ECM accumulation within the TM cells [51]. Inhibition of ATX, LPA receptors, or ROCK all suppressed the dexamethasone-induced fibrotic changes and cytoskeletal reorganization, indicating that ROCK inhibitors represent a potential anti-fibrotic therapeutic target for steroid responders.

## 5. Neuroprotective Potential of Rho Kinase Inhibitors

ROCK inhibition has demonstrated neuroprotective effects in preclinical models by reducing pro-apoptotic markers and increasing pro-survival factors, particularly in the context of retinal ganglion cell (RGC) survival. In an optic nerve crush model, Koch et al. [52] reported that ROCK2 knockdown reduced pro-apoptotic markers (e.g., calpain and caspase-3), while enhancing autophagic flux and increasing levels of phosphorylated Akt and collapsin response mediator protein 2, which are key pro-survival proteins. Not only was RGC survival promoted by ROCK inhibition, but axonal degeneration was also attenuated and regeneration beyond the crush site was facilitated. Similar findings were demonstrated by two studies using rat models, which showed that expression of the anti-apoptotic gene B-cell lymphoma/leukemia-2 (*Bcl-2*) and the Bcl-2 protein were increased by ROCK inhibitors, while caspase-3 levels were reduced [53,54].

In addition to modulating apoptotic and autophagic pathways, ROCK inhibitors have been shown to exert anti-inflammation and antioxidant effects. Changes in microglial activation patterns, decreased production of inflammatory cytokines (TNF and IL-1α) [55,56], and attenuation of reactive oxygen species (ROS) levels [57,58,59] were reported. These anti-neuroinflammation and antioxidant properties also contribute to the overall neuroprotective profile of ROCK inhibitors (Table 2).

Multiple compounds, including AR-13324, K-115, fasudil, and Y-27632, have demonstrated efficacy in neuroprotection in models of optic nerve injury or OHT. RGC survival was significantly improved and robust axonal regeneration was promoted by topical administration of AR-13324, likely through the inhibition of downstream ROCK targets such as cofilin and LIM domain kinase (LIMK) in both retinal and optic nerve tissues [58]. K-115 was shown to attenuate oxidative stress-induced RGC death by reducing oxidation of lipids and ROS production [57]. Moreover, Yamagishi-Kimura et al. [60] reported significant protection of RGC from NMDA-induced RGC loss following treatment with 100 μM ripasudil in mice. This protective effect was attributed to the suppression of oxidative stress markers by ripasudil, including reductions in glutathione levels and calpain activity. These findings suggest that the neuroprotective effects of ripasudil involve multiple antioxidative mechanisms, including reductions in lipid oxidation, ROS production, glutathione levels, and calpain activity. Ganglion cell loss was reduced and inner plexiform thickness was preserved against glutamate-related excitotoxicity by intravitreal injection of fasudil [61]. RGC viability was enhanced by Y-27632 in both in vitro and in vivo systems, likely by stimulating pro-survival pathways such as Akt [62].

Although these findings are promising, the preclinical nature of most studies limits their direct clinical applicability. Future research should aim to define optimal dosing regimens, evaluate long-term safety profiles, and better elucidate how these neuroprotective mechanisms interact in various types of optic nerve injury.

## 6. Limitation of Rho Kinase Inhibitors

### 6.1. Ocular Adverse Effect

Conjunctival hyperemia is the most frequently reported side effect associated with ROCK inhibitors, with incidence rates ranging from 37% to 65%—notably higher than those for timolol (8–14%) and latanoprost (9–22%) [63]. It is proposed that hyperemia is caused by the vasodilation effect of ROCK inhibitors in vascular smooth muscle of the conjunctiva. The conjunctival hyperemia is self-limited and transient. Peak occurrence typically occurs at 10–15 min post-instillation and resolution is observed within 1–2 h [64]. However, treatment adherence may still be reduced by this side effect. In one study, 7.1% of patients discontinued fixed-dose netarsudil/latanoprost due to hyperemia [65]. A reduction in conjunctival hyperemia has been reported with novel agents. In animal models, AMA0076 was associated with a decreased incidence of hyperemia, potentially due to its rapid metabolic inactivation on the ocular surface [66]. In a crossover randomized clinical trial, the severity of hyperemia was significantly reduced in the group receiving the fixed-dose combination of ripasudil and brimonidine compared to the ripasudil monotherapy group. This attenuation is presumed to be mediated by the vasoconstrictive effect of brimonidine, which may counteract the vasodilatory response induced by ROCK inhibition [67].

Another notable adverse effect of ROCK inhibitors involves the cornea. Corneal verticillata has been reported in 13–26% of patients receiving ROCK inhibitors [63], occurring more frequently in those treated with netarsudil than with ripasudil [63,68]. This phenomenon was absent in non-Rho kinase inhibitor arms in clinical trials. Reticular epithelial corneal edema is less common than corneal verticillata but remains clinically relevant. Schlötzer-Schrehardt et al. [69] demonstrated that several genes involved in corneal epithelial junctions were downregulated by netarsudil, leading to impaired epithelial barrier function. This may represent a pathophysiological mechanism underlying the development of reticular epithelial corneal edema. Notably, these drug-specific effects were reversible upon discontinuation, emphasizing the dynamic nature of corneal epithelial responses to ROCK inhibition [69,70].

### 6.2. Modest IOP Reduction Efficacy

Current clinical evidence does not support the use of netarsudil or ripasudil as an optimal first-line monotherapy for POAG or OHT. Although 0.02% netarsudil has demonstrated non-inferiority to 0.5% timolol or bimatoprost 0.01% in monotherapy trials [21,23,71], a meta-analysis conducted by Lee et al. found that latanoprost monotherapy resulted in significantly greater IOP reduction compared to netarsudil after 4 to 6 weeks of treatment [72]. Furthermore, among ROCK inhibitors, a greater mean IOP-lowering effect was shown by netarsudil 0.02% than ripasudil 0.4%, with a statistically significant difference of 1.74 mmHg being observed [14]. These findings suggest that while ROCK inhibitors may serve as useful adjunctive therapies, their routine use as standalone first-line treatments for POAG or OHT is not supported by current data.

In combination regimens, no significant difference in IOP reduction was observed between the netarsudil–latanoprost fixed combination and the bimatoprost–timolol combination [73]. Despite their unique mechanism of action, ROCK inhibitors have yet to demonstrate a clear advantage over existing therapies in terms of efficacy.

## 7. Prospects for Rho Kinase Inhibitors

Concerns regarding ROCK inhibitors are focused on their relatively limited IOP-lowering efficacy and their association with ocular side effects. The conventional aqueous humor outflow pathway is enhanced by ROCK inhibitors, specifically through modulation of the TM. The function of TM outflow involves a complex interplay of structural components and biochemical mediators, with the contractile state of TM cells playing a pivotal role. The actin cytoskeleton, which maintains the basal tone of TM cells, is regulated in part by ROCK signaling [74].

Nonetheless, ROCK is only one component of the broader regulatory landscape governing TM dynamics. Other signaling molecules and enzymes—such as TGF-β, nitric oxide (NO), and adenosine triphosphate (ATP)—are also known to influence TM behavior and outflow resistance [74]. The limited efficacy of ROCK inhibitors as monotherapy may therefore be attributed to the complexity of this regulatory environment.

To enhance IOP-lowering efficacy, dual-action compounds that target ROCK in conjunction with other key signaling pathways have been investigated by recent studies. For example, a dual kinase inhibitor targeting both ROCK and myosin light chain kinase (ROCK/MYLK) demonstrated greater reduction in IOP compared to netarsudil in both rabbit models of OHT and normotensive non-human primates [75,76]. Another promising approach involves the dual inhibition of ROCK and LIMK, which has been shown to increase TM relaxation and aqueous outflow, with the added potential of fewer side effects due to improved selectivity [77]. Furthermore, ongoing clinical research is focused on developing novel ROCK inhibitors with improved efficacy and safety profiles compared to currently marketed agents [78].

## 8. Conclusions

ROCK inhibitors represent a mechanistically novel and pharmacologically versatile class of agents in the treatment of glaucoma. By targeting cytoskeletal dynamics and ECM remodeling in the TM, they offer a distinct mechanism of action that complements existing ocular hypotensive therapies. In addition to reducing IOP, ROCK inhibitors contribute to neuroprotection, enhance ocular perfusion, and modulate postoperative fibrosis and inflammation, broadening their potential therapeutic role. Nonetheless, their current use as first-line monotherapy is limited by their modest IOP-lowering efficacy and notable ocular side effects. The emergence of dual kinase inhibitors with improved target selectivity and enhanced efficacy underscores the need for further research. In preclinical studies, superior IOP reduction compared to pure ROCK inhibitors has been demonstrated with dual kinase inhibitors through the modulation of additional cytoskeletal pathways. Moreover, the potential for fewer side effects has been noted, likely attributable to enhanced target specificity. Future clinical applications of these dual kinase inhibitors appear promising. Patients with inadequate IOP control despite maximum medical therapy, or those who experience intolerable side effects from existing treatments, may particularly benefit from dual kinase inhibitors. As our understanding of the molecular regulators of aqueous outflow and optic nerve health is deepened, the landscape of glaucoma management may be reshaped by the development of next-generation ROCK-based therapies.

## Figures and Tables

**Table 1:** Modulation of postoperative inflammation and fibrosis following trabeculectomy.

Therapy	Agent	Mechanism	Cellular and Clinical Effect	Authors
Conventional therapy	Mitomycin-C	Mitomycin-C is an alkylating agent that inhibits cellular proliferation.	Suppresses fibroblast proliferation and inhibits collagen production; significantly lowers IOP in primary trabeculectomy.	Jampel et al. [39] Bindlish et al. [40]
	5-Fluorouracil	5-Fluorouracil inhibits thymidylate synthase and further halts DNA synthesis in replicating cells.	Inhibit human Tenon’s fibroblast proliferation; improves IOP control after trabeculectomy.	Khaw et al. [42] Rothman et al. [43]
ROCK ^1^ inhibitor	AR12286	Inhibits TGF ^2^-β1-induced myofibroblast transdifferentiation, as well as fibronectin and collagen production.	Inhibits conjunctival fibroblast activation; effective at lower concentrations compared to other ROCK inhibitors for antifibrotic effects.	Cheng et al. [45]
	Y-27632	Inhibits TGF-β1/Smad2,3 signaling, suppressing α-SMA ^3^, CTGF ^4^, fibroblast activation, and ECM ^5^ production.	Inhibits profibrotic signaling in tenon’s fibroblasts; effective in vitro in fibroblast cultures.	Feng et al. [46]

^1^ ROCK, Rho kinase; ^2^ TGF, Transforming growth factor; ^3^ α-SMA, α-Sigma-Aldrich; ^4^ CTGF, Connective tissue growth factor; ^5^ ECM, Extracellular matrix.

**Table 2:** Primary neuroprotective mechanisms of Rho kinase inhibitors.

Mechanism	Molecular Mediators	Cellular Effects	Clinical Relevance	Authors
Anti-apoptotic Signaling	Downregulation: caspase-3, calpain Upregulation: Bcl-2 ^1^, pAkt ^2^, CRMP2 ^3^	Reduced cell death, increased cell survival	Potential to prevent retinal cell loss in various degenerative conditions	Koch et al. [52] Wang et al. [53] Zhang et al. [54]
Inflammatory Modulation	Downregulation: TNF ^4^, IL-1α ^5^	Reduced microglial activation, decreased inflammatory cytokine production	Alleviate chronic inflammation associated with retinal degeneration	Wen et al. [55] Sato et al. [56]
Oxidative Stress Response	Downregulation: ROS ^6^	Protection against oxidative damage, improved cell survival	Mitigate oxidative stress-induced retinal damage in various pathologies	Yamamoto et al. [57] Shaw et al. [58] Quillen et al. [59]

^1^ Bcl-2, B-cell lymphoma/leukemia-2; ^2^ pAkt, phospho-Akt; ^3^ CRMP2, collapsin response mediator protein 2; ^4^ TNF, tumor necrosis factor; ^5^ IL-1α, interleukin-1α; ^6^ ROS, reactive oxygen species.

## Data Availability

Not applicable.

## Abbreviations

The following abbreviations are used in this manuscript:

ATP	Adenosine triphosphate
ATX	Autotaxin
Bcl-2	B-cell lymphoma/leukemia-2
CTGF	Connective tissue growth factor
ECM	Extracellular matrix
IL	Interleukin
IOP	Intraocular pressure
LIMK	LIM domain kinase
LPA	Lysophosphatidic acid
NO	Nitric oxide
OHT	Ocular hypertension
q.d.	Once daily
POAG	Primary open angle glaucoma
ROS	Reactive oxygen species
RGC	Retinal ganglion cell
ROCK	Rho kinase
TM	Trabecular meshwork
TGF-β	Transforming growth factor-β
TNF	Tumor necrosis factor
b.i.d.	Twice daily
